# Multi-omic approach provides insights into osmoregulation and osmoconformation of the crab *Scylla paramamosain*

**DOI:** 10.1038/s41598-020-78351-w

**Published:** 2020-12-10

**Authors:** Jiaojiao Niu, Xue Lei Hu, Jack C. H. Ip, Ka Yan Ma, Yuanyuan Tang, Yaqin Wang, Jing Qin, Jian-Wen Qiu, Ting Fung Chan, Ka Hou Chu

**Affiliations:** 1 Simon F. S. Li Marine Science Laboratory, School of Life Sciences, The Chinese University of Hong Kong, Shatin, N.T., Hong Kong, China; 2 Department of Biology, Hong Kong Baptist University, Kowloon Tong, Kowloon, Hong Kong, China; 3 School of Pharmaceutical Sciences (Shenzhen), Sun Yat-Sen University, Guangzhou, 510275 China; 4 State Key Laboratory of Agrobiotechnology, School of Life Sciences, The Chinese University of Hong Kong, Shatin, N.T., Hong Kong, China

**Keywords:** Molecular biology, Zoology

## Abstract

Osmoregulation and osmoconformation are two mechanisms through which aquatic animals adapt to salinity fluctuations. The euryhaline crab *Scylla paramamosain,* being both an osmoconformer and osmoregulator, is an excellent model organism to investigate salinity adaptation mechanisms in brachyurans. In the present study, we used transcriptomic and proteomic approaches to investigate the response of *S. paramamosain* to salinity stress. Crabs were transferred from a salinity of 25 ppt to salinities of 5 ppt or 33 ppt for 6 h and 10 days. Data from both approaches revealed that exposure to 5 ppt resulted in upregulation of ion transport and energy metabolism associated genes. Notably, acclimation to low salinity was associated with early changes in gene expression for signal transduction and stress response. In contrast, exposure to 33 ppt resulted in upregulation of genes related to amino acid metabolism, and amino acid transport genes were upregulated only at the early stage of acclimation to this salinity. Our study reveals contrasting mechanisms underlying osmoregulation and osmoconformation within the salinity range of 5–33 ppt in the mud crab, and provides novel candidate genes for osmotic signal transduction, thereby providing insights on understanding the salinity adaptation mechanisms of brachyuran crabs.

## Introduction

Osmoconformation and osmoregulation are two mechanisms that aquatic animals adopt to cope with osmotic perturbations in the environment. Most marine invertebrates and some vertebrates (e.g., sharks, skates and hagfish) osmoconform by using organic osmolytes to keep osmotic pressure of body fluids equal to that of the external environment. Organic osmolytes do not perturb cellular macromolecules but instead protect macromolecules from denaturation^1^, which is the main reason why they are utilized in osmoconformation. There are many kinds of organic osmolytes employed by different organisms^1^. In general, elasmobranchs’ primary organic osmolytes are urea and trimethylamine oxide^2,3^, while most marine invertebrates mainly use free amino acids and methylamines as organic osmolytes^4^. In contrast to osmoconformation, other organisms osmoregulate, maintaining the osmotic pressure of their body fluids at levels different from that of the environment. It is widely known that most vertebrates and freshwater/estuarine invertebrates are osmoregulators, which display a differing array of osmoregulatory organs and mechanisms. For example, bony fishes osmoregulate using kidneys, gills and gut^5^, while gills and antennal glands are the primary osmoregulatory tissues in crustaceans^6^. Nevertheless, some ion transporters and ion channels, such as V-type H^+^ ATPase, Na^+^/K^+^ ATPase, Na^+^ channel, Na^+^-K^+^-2Cl^−^ cotransporter, and Cl^-^/HCO_3_^-^ exchanger contribute to osmoregulation in both fishes and crustaceans^5–7^.

As a group of crustaceans, brachyuran crabs widely distribute in waters of different salinities, including freshwater, marine, estuarine and intertidal habitats. Accordingly, different salinity adaptation strategies are found in crabs from different habitats, including both osmoconformation and osmoregulation. In details, freshwater crabs (e.g., *Dilocarcinus pagei*) can osmoregulate via active ion transport^6–8^. It is proposed that active salt absorption in the gills of freshwater crabs is accomplished via a suite of ion transporters and supporting enzymes: Na^+^ absorption occurs via a combination of apical Na^+^ channel and V-type H^+^ ATPase, and the basolateral Na^+^/K^+^ ATPase, while Cl^−^ absorption is accomplished via apical co-transport, Cl^−^/HCO_3_^−^ exchanger and basolateral Cl^−^ channels^6^. By contrast, marine crabs are osmoconformers (e.g., *Macropipus puber*^9^ and *Hepatus pudibundus*^10^), and use mainly free amino acids as organic osmolytes^4,7^. Yet studies on their osmoconforming mechanism are rare. Besides, different from freshwater and marine crabs that can merely tolerate very small fluctuation in environmental salinity, euryhaline crabs by definition can adapt to environments with a wide range of salinities (e.g. estuarine and intertidal zones). More importantly, some euryhaline crabs exhibit both osmoregulation and osmoconformation^9,11^, such as *Scylla* spp. and *Carcinus maenas*. Thus, these euryhaline crab species are good model organisms in investigations to explore the osmoregulation and osmoconformation mechanisms of crabs.

In fact, a number of studies on salinity adaptation of euryhaline crabs have been conducted, showing the involvement of ion transport, amino acid metabolism, and energy metabolism in the process^12–17^. However, these studies have two main limitations. First, the molecular mechanisms underlying osmoconformation have rarely been investigated. Some amino acid metabolism genes and pathways showed differential gene expression in response to salinity change^15,17,18^, but whether the crabs use them to osmoconform or osmoregulate remains unknown. Secondly, studies addressing osmotic signal transduction in crabs are limited. Signal transduction is a fundamental aspect of osmoregulation, in which various signaling molecules (e.g. mitogen-activated protein kinase (MAPK) cascades, transcription factors and hormones) are stimulated by osmolality changes, and then regulate ion transport or other effectors^19–21^. Although several studies mention osmotic signal transduction, they each discuss different genes. For example, integrin was reported to be involved in hyper-osmoregulation of *C. maenas*, while crustacean hyperglycemic hormone was reported as a mediator of osmotic signal transduction in *Portunus trituberculatus*^13,16^. The unstructured nature of literature on crab osmoregulation suggests that a comprehensive investigation on osmoregulation and osmoconforming mechanisms of brachyuran crabs is still pending.

The mud crab *Scylla paramamosain* is distributed along the coast of the South China Sea^11^. Because of its abundance, fast growth rate and high market value, the species is an important aquaculture crab in China. *S. paramamosain* inhabits waters of salinity around 5 ~ 33 ppt^22^. It is an osmoconformer in high salinity (around 25 ~ 45 ppt) environments but osmoregulator in low salinity (e.g., 5 ppt) environments^11^, and thus could serve as a good model to investigate salinity adaptation mechanisms in the Brachyura. In the present study, *S. paramamosain* individuals were exposed to three salinities (5, 25 and 33 ppt) for a short (6 h) and a long (10 days) period, and genes differentially expressed in gills were profiled at transcriptional and translational levels, using high-throughput next-generation sequencing and mass spectrometry, respectively. This work may yield some valuable information for future research on salinity adaptation mechanisms in the brachyuran crabs.

## Results

**Figure 1:**
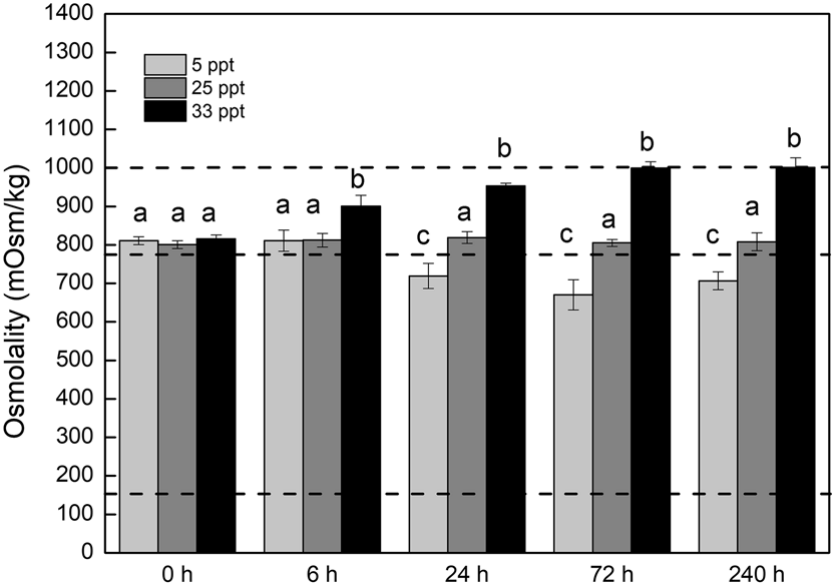
Hemolymph osmolality of crabs during different salinity acclimation. Dash lines represent osmolality of 5 ppt, 25 ppt and 33 ppt seawater (from bottom up). Significant differences are indicated by letters (*p* < 0.05, one-way ANOVA followed by Dunnett’s multiple comparison).

The hemolymph osmolality of *S. paramamosain* in the 25 ppt control group was maintained at around 800 mmol/kg throughout the experimental period (Fig. 1). During 33 ppt salinity acclimation, the hemolymph osmolality of the treatment group became significantly higher than that of the control group at 6 h (One-way ANOVA, *p* < 0.05), and continued to increase to about 1000 mmol/kg (the same concentration as the external medium). On the other hand, the hemolymph osmolality decreased to 700 mmol/kg one day after transfer to 5 ppt and maintained a similar level afterward. These results show that the mud crabs were able to keep their hemolymph osmolality similar to that of the environment when exposed to 33 ppt but that the osmolality was significantly higher than the environment when the crabs were exposed to 5 ppt (One-way ANOVA, *p* < 0.05).

### Overview of transcriptome data and differentially expressed transcripts

Crabs in 25 ppt (control group), exposed to 5 ppt for 6 h (5 ppt/6 h group), exposed to 5 ppt for 10 days (5 ppt/10 days group), exposed to 33 ppt for 6 h (33 ppt/6 h group), and exposed to 33 ppt for 10 days (33 ppt/10 days group) were sampled for transcriptomic and proteomic analyses. A total of 104.9 Gb high quality (Q30 > 94%) clean raw sequencing data were obtained from a total of 15 samples (triplicate per group), with each sample having more than 30 M reads. The Trinity assembly produced more transcripts than the BinPacker assembly, albeit transcripts produced by BinPacker had a higher N50 and average length (Supplementary Table S1). More than 96% completeness was achieved with both assembly methods, although 17 more complete BUSCOs were identified in the BinPacker assembly. Assembly merging by Transfuse resulted in more transcripts compared to the standalone BinPacker assembly, as well as improved N50 and average length compared to the Trinity assembly. However, the completeness of the transcriptome was not improved by Transfuse. Therefore, the assembly generated from Transfuse was used for subsequent analyses. A total of 68,564 coding sequences were identified, of which 44,654 showed significant BLASTP hits to known proteins in the UniProt database, and 41,901 could be annotated in the Gene Ontology (GO) database. There were 3326, 618, 422 and 310 significantly differentially expressed transcripts (DETs) in the groups 5 ppt/6 h, 5 ppt/10 days, 33 ppt/6 h and 33 ppt/10 days, respectively (Supplementary Table S2). The two 5 ppt groups had more DETs than the 33 ppt groups, and the groups after 6-h exposure had more DETs than those under 10-days exposure. The 5 ppt/6 h group had the most (2883) unique DETs, while the 33 ppt/6 h group had the fewest (214) unique DETs (Supplementary Fig. S1A). The 5 ppt/6 h group shared most DETs with the other three groups, and the 33 ppt/10 days group shared the fewest DETs with the other three groups.

**Figure 2:**
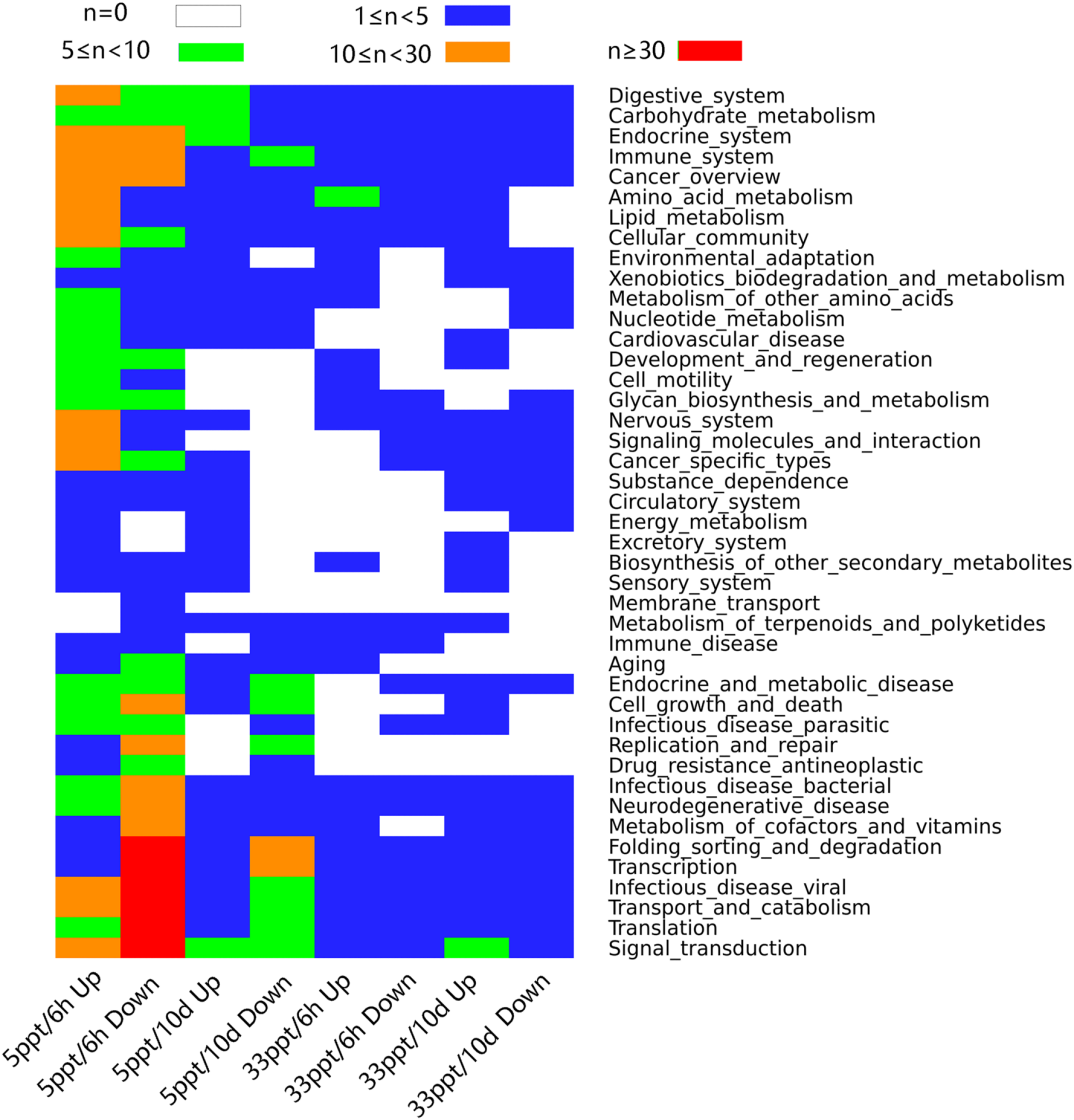
Heat map diagram of KEGG annotation of differentially expressed transcripts in each treatment group using gplots package in R. Different colors indicate the number of transcripts (n).

Annotation with KEGG orthology database showed that a high proportion of DETs of the 5 ppt/6 h group were involved in genetic information processing (Fig. 2), including translation (66), folding, sorting and degradation (63), transcription (40), and replication and repair (21). Most of them were downregulated. The functional categories of signal transduction and endocrine system contained 27 and 21 upregulated DETs respectively, which may mediate osmotic signal transduction. All four energy metabolism related DETs showed increased expression in the 5 ppt/6 h group. In the 5 ppt/10 days group, the top three functional categories with the most upregulated transcripts were digestive system (8), endocrine system (6) and signal transduction (6). In the 33 ppt/6 h group, eight DETs, seven of which were upregulated, were involved in amino acid metabolism, suggesting that this process was activated during high salinity acclimation. Besides, the categories of translation, signal transduction, transport and catabolism, and infectious disease: viral had most DETs. In the 33 ppt/10 days group, the top three functional categories with most upregulated transcripts were signal transduction, amino acid metabolism, and cell growth and death. All the four DETs involved in amino acid metabolism were upregulated. For upregulated DETs in the two 33 ppt groups, no GO term was significantly enriched. Upregulated DETs in the two 5 ppt groups were mainly enriched in ion transport and anion transport (Supplementary Table S3). For downregulated DETs in the 5 ppt/10 days, 5 ppt/6 h and 33 ppt/6 h groups, enriched GO terms were not directly related to osmoregulation. Downregulated DETs in the 33 ppt/10 days group were enriched in chloride transport and sulfate transmembrane transporter activity. Abundances of DETs involved in functional categories enriched (ion transport) and containing most differentially expressed genes (signal transduction, endocrine system, energy metabolism and amino acid metabolism) are presented in Supplementary Fig. S2.

Based on previous studies on fishes and crustaceans^6,19–21,23^, part of the DETs in the two 5 ppt groups were investigated in detail (Table 1). Ten ion transport gene transcripts were upregulated in these groups, including V-type proton ATPase subunits A, B and D2, sodium-dependent phosphate transport protein 2A, sodium bicarbonate transporter-like protein 11, chloride channel protein 2, G protein-activated inward rectifier potassium channel 4, carbonic anhydrase 2, sulfate transporter, and solute carrier family 26 member 6, while aquaporin-12A (AQ12A) was downregulated (Table 1). Ten genes involved in osmoregulatory signal transduction were upregulated in the 5 ppt/6 h group, such as focal adhesion kinase 1 (FAK1), integrin alpha-V (ITAV), myosin heavy chain (MYSN) and signaling mucin HKR1 (Table 1). Five genes involved in energy metabolism (e.g., inorganic pyrophosphatase and NADH dehydrogenase 1 beta subcomplex subunit 10) showed upregulated expression in the two 5 ppt groups. Two stress response genes (endoplasmin and 60 kDa heat shock protein) showed elevated expression in the 5 ppt/6 h group. In the two 33 ppt groups, 10 DETs that are likely to participate in osmoconformation in mud crabs, were investigated in detail (Table 2). Two amino acid transport (excitatory amino acid transporter 3 and mitochondrial basic amino acids transporter) genes showed increased expression in the 33 ppt/6 h group. Seven amino acid metabolism genes (e.g., glutamate dehydrogenase and aspartate aminotransferase) were upregulated in the two groups, while only one gene (ODB2) was downregulated in the 33 ppt/6 h group. Real time qPCR results were consistent with transcriptomic results (Supplementary Fig. S3).

**Table 1:** Log2 fold change of differentially expressed transcripts of interest in 5 ppt groups.

Gene ID	Gene name	5 ppt/6 h	5 ppt/10 days
**Ion transport**			
AQ12A	Aquaporin-12A	**− 7.18**	**− 6.15**
NPT2A	Sodium-dependent phosphate transport protein 2A	**3.42**	**3.17**
S4A11	Sodium bicarbonate transporter-like protein 11	**3.66**	3.21
VATA	V-type proton ATPase catalytic subunit A	**1.64**	0.88
VATB	V-type proton ATPase subunit B	**1.78**	1.64
VATD2	V-type proton ATPase subunit D 2	**1.38**	1.05
CLCN2	Chloride channel protein 2	**3.98**	**3.16**
KCNJ5	G protein-activated inward rectifier potassium channel 4	**1.45**	0.26
CAH2	Carbonic anhydrase 2	3.4	**7**
S26A2	Sulfate transporter	1.6	**3.23**
S26A6	Solute carrier family 26 member 6	1.36	**2.99**
**Signal transduction**			
FAK1	Focal adhesion kinase 1	**3.22**	2.69
ITAV	Integrin alpha-V	**2.66**	0.56
MYSN	Myosin heavy chain, non-muscle	**1.94**	0.24
PPB	Alkaline phosphatase	**3.04**	3.66
EI2BA	Translation initiation factor eIF-2B subunit alpha	**− 2.38**	0.54
EI2BB	Translation initiation factor eIF-2B subunit beta	**− 1.71**	0.78
HKR1	Signaling mucin HKR1	**3.79**	**− **0.03
MYPT1	Protein phosphatase 1 regulatory subunit 12A	**1.03**	0.25
PR15A	Protein phosphatase 1 regulatory subunit 15A	**− 0.82**	0
ANK3	Ankyrin-3	8.73	**5.7**
**Energy metabolism**			
SERC	Probable phosphoserine aminotransferase	**8.31**	0.1
IPYR	Inorganic pyrophosphatase	**4.68**	**2.2**
NDUBA	NADH dehydrogenase 1 beta subcomplex subunit 10	**1.22**	0.81
THIL	Acetyl-CoA acetyltransferase	**9.05**	**11.43**
SDHB	Succinate dehydrogenase [ubiquinone] iron-sulfur subunit	**− **0.64	**11.01**
**Stress response**			
CH60	60 kDa heat shock protein	**1.7**	**− **0.18
ENPL	Endoplasmin	**2.01**	0.04

Significant differences (FDR < 0.001) are in bold.

**Table 2:** Log2 fold change of differentially expressed transcripts of interest in 33 ppt groups.

Gene ID	Gene name	33 ppt/6 h	33 ppt/10 days
EAA3	Excitatory amino acid transporter 3	**2.38**	2.36
MCATL	Mitochondrial basic amino acids transporter	**1.1**	**− **1.6
AATC	Aspartate aminotransferase, cytoplasmic	**3.59**	**3.32**
CDO1	Cysteine dioxygenase type 1	**2.03**	**− **1.06
ODB2	Lipoamide acyltransferase component of branched-chain alpha-keto acid dehydrogenase complex	**− 1.18**	0.62
FTCD	Formimidoyltransferase-cyclodeaminase	**1.82**	**2.48**
ECHM	Enoyl-CoA hydratase, mitochondrial	**3.25**	**− **0.92
AASS	Alpha-aminoadipic semialdehyde synthase	**1.25**	**− **1.51
DHE3	Glutamate dehydrogenase	**1.91**	**1.77**
GLNA	Glutamine synthetase	**1.35**	**2.08**

Significant differences (FDR < 0.001) are in bold.

### Overview of proteome data and differentially expressed proteins

A total of 65,059 peptide sequences and 1957 protein groups were identified (FDR < 0.01). The correlation between the expression level of transcripts and proteins in all experimental groups is low (Pearson’s R < 0.3, Spearman’s R < 0.3, linear correlation coefficient R^2^ < 0.1; Supplementary Fig. S4). A total of 31 genes showed differential expression in both transcriptomic and proteomic data (Supplementary Table S4) with more of them being upregulated than downregulated. The 5 ppt/6 h group had the most genes showing differential expression at both transcriptional and translational levels. Some of these genes are directly associated with osmoconformation or osmoregulation (e.g., V-type proton ATPase subunit B, integrin alpha-V and carbonic anhydrase 2), while others are mainly related to carbohydrate metabolism, genetic information processing and cytoskeleton organization.

**Figure 3:**
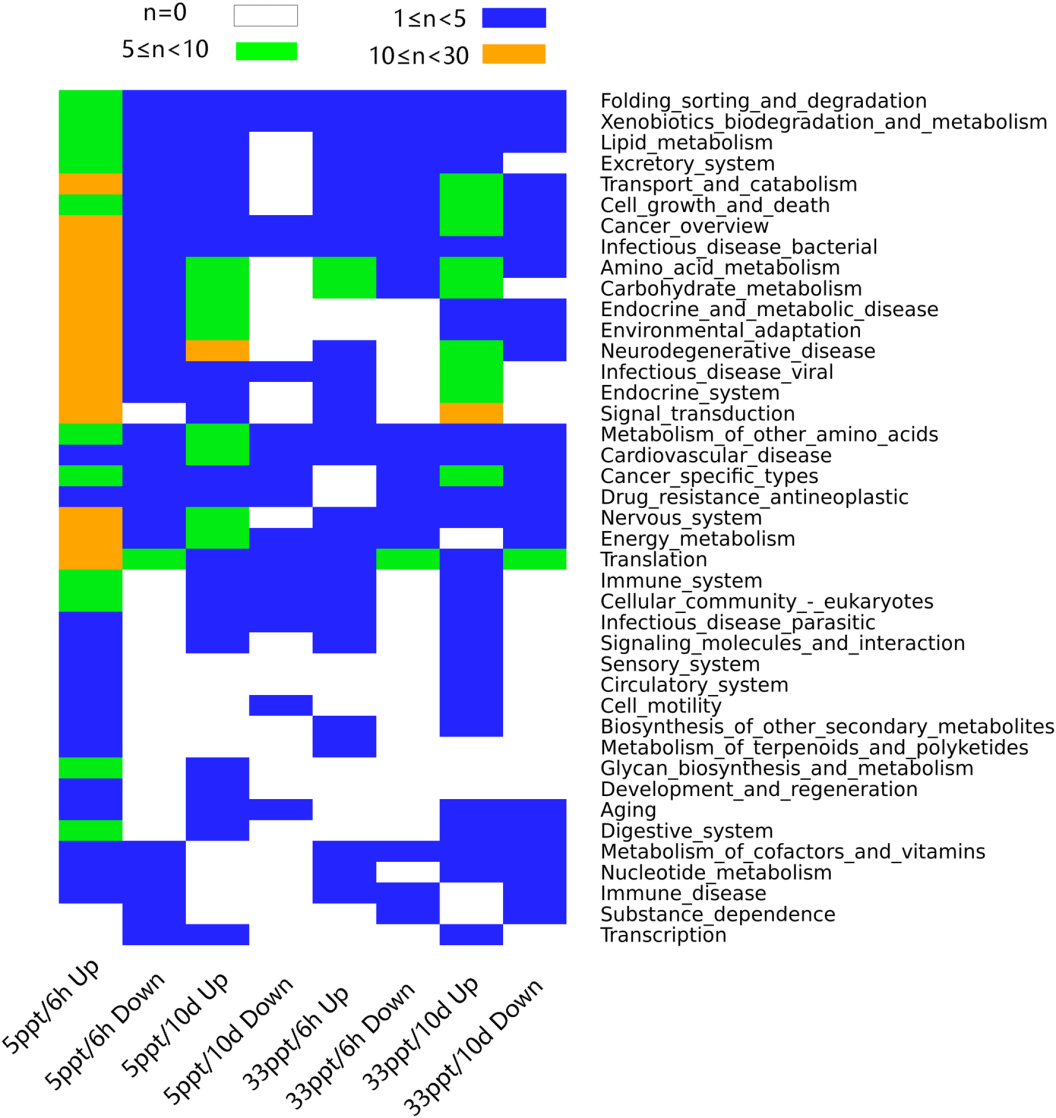
Heat map diagram of KEGG annotation of differentially expressed proteins in each treatment group using gplots package in R. Different colors indicate the number of proteins (n).

There were 260, 91, 60, 140 differentially expressed proteins (DEPs) in the 5 ppt/6 h, 5 ppt/10 days, 33 ppt/6 h and 33 ppt/10 days treatment groups respectively (Supplementary Table S2). All four groups had more upregulated DEPs than downregulated DEPs. Only five DEPs were shared by all four treatments. The 5 ppt/6 h group had the most (177) unique DEPs, while the 33 ppt/6 h group had the fewest (22) (Supplementary Fig. S1B). DEPs upregulated in the two 5 ppt groups were mainly involved in energy metabolism, amino acid metabolism, carbohydrate metabolism and environmental adaptation (Fig. 3). In the 5 ppt/6 h group, signal transduction, nervous system and endocrine system also contained a large number of upregulated proteins. In the two 33 ppt groups, the top three function categories with the most upregulated proteins were amino acid metabolism, carbohydrate metabolism and signal transduction. Abundances of DEPs associated with ion transport, signal transduction, endocrine system, energy metabolism and amino acid metabolism are presented in Supplementary Fig. S2.

Based on previous studies on fishes and crustaceans^6,19–21,23^, part of the DEPs in the two 5 ppt groups were investigated in detail (Table 3). Seven DEPs were associated with ion transport, of which V-type proton ATPase subunits B, C and D2 as well as chloride channel protein 2, carbonic anhydrase 2, and ammonium transporter Rh type B were upregulated, while carbonic anhydrase 7 was downregulated. Six DEPs related to osmotic signal transduction showed increased expression in the 5 ppt/6 h group (e.g., integrin alpha-V, myosin regulatory light chain sqh and mitogen-activated protein kinase 14). Almost all the energy metabolism related DEPs were upregulated in the 5 ppt groups, except for NADH dehydrogenase [ubiquinone] 1 alpha subcomplex subunit 13, which showed decreased expression in the 5 ppt/6 h group. Two heat shock proteins (heat shock cognate 71 kDa protein and 60 kDa heat shock protein) were upregulated in the 5 ppt/6 h group. Fifteen DEPs of the two 33 ppt groups were associated with amino acid metabolism (Table 4), most of which (e.g., glutamate dehydrogenase and aldehyde dehydrogenase) showed increased expression in the two groups, but 3-hydroxyisobutyrate dehydrogenase and acylpyruvase showed decreased expression in the 33 ppt/10 days group.

**Table 3:** Log2 fold change of differentially expressed proteins of interest in 5 ppt groups.

Gene ID	Gene name	5 ppt/6 h	5 ppt/10 days
**Ion transport**			
VATB	V-type proton ATPase subunit B	**1.2**	**2.38**
VATC	V-type proton ATPase subunit C	**2.14**	**2.74**
VATD2	Probable V-type proton ATPase subunit D 2	**1.93**	**3.53**
CAH2	Carbonic anhydrase 2	1.21	**2.59**
CAH7	Carbonic anhydrase 7	**− 2.72**	**− 5.32**
CLCN2	Chloride channel protein 2	1.26	**1.39**
RHBG	Ammonium transporter Rh type B	**1.87**	1.36
**Signal transduction**			
ITAV	Integrin alpha-V	**2.96**	**2.85**
MK14	Mitogen-activated protein kinase 14	**Inf**	**Inf**
SQH	Myosin regulatory light chain sqh	**2.54**	2.44
ANK3	Ankyrin-3	**4.7**	2.96
CALR	Calreticulin	**Inf**	0
PPB	Alkaline phosphatase	**Inf**	Inf
**Energy metabolism**			
AT5F1	ATP synthase subunit b, mitochondrial	**2.96**	1.61
ATPB	ATP synthase subunit beta, mitochondrial	**2.61**	1.37
ATPG	ATP synthase subunit gamma, mitochondrial	**1.7**	1.83
QCR2	Cytochrome b-c1 complex subunit 2, mitochondrial	**3.45**	0.69
QCR7	Cytochrome b-c1 complex subunit 7	**3.88**	3.08
IPYR	Inorganic pyrophosphatase	**2.27**	**2.51**
NDUAA	NADH dehydrogenase [ubiquinone] 1 alpha subcomplex subunit 10	0	**Inf**
NDUAD	NADH dehydrogenase [ubiquinone] 1 alpha subcomplex subunit 13	**− Inf**	**Inf**
NDUB8	NADH dehydrogenase [ubiquinone] 1 beta subcomplex subunit 8	**2.98**	1.78
NDUB9	NADH dehydrogenase [ubiquinone] 1 beta subcomplex subunit 9	**Inf**	**Inf**
NDUC2	NADH dehydrogenase [ubiquinone] 1 subunit C2	1.93	**3.97**
NDUV1	NADH dehydrogenase [ubiquinone] flavoprotein 1	**Inf**	Inf
NDUV2	NADH dehydrogenase [ubiquinone] flavoprotein 2	**0.93**	0.96
NDUS2	NADH dehydrogenase [ubiquinone] iron-sulfur protein 2	**1.13**	0.97
NDUS7	NADH dehydrogenase [ubiquinone] iron-sulfur protein 7	**2.33**	1.7
NDUS1	NADH-ubiquinone oxidoreductase 75 kDa subunit	**1.89**	0.89
ATPK	Putative ATP synthase subunit f, mitochondrial	**2**	1.43
SDHA	Succinate dehydrogenase [ubiquinone] flavoprotein subunit	**3.01**	2.29
**Stress response**			
HSP7C	Heat shock cognate 71 kDa protein	**Inf**	0
CH60	60 kDa heat shock protein, mitochondrial	**2.1**	3.01

Significant differences (FDR < 0.05) are in bold.

**Table 4:** Log2 fold change of differentially expressed proteins of interest in 33 ppt groups.

Gene ID	Gene name	33 ppt/6 h	33 ppt/10 days
ALDH2	Aldehyde dehydrogenase, mitochondrial	**3.29**	2.72
OAT	Ornithine aminotransferase, mitochondrial	**2.31**	1.62
FTCD	Formimidoyltransferase-cyclodeaminase	**2.59**	**3.01**
ECHM	Enoyl-CoA hydratase, mitochondrial	**1.55**	**1.97**
3HIDH	3-Hydroxyisobutyrate dehydrogenase	**− **0.28	**− Inf**
FAHD1	Acylpyruvase	**− **0.94	**− Inf**
AASS	Alpha-aminoadipic semialdehyde synthase	0.98	**2.13**
ARLZ	Probable argininosuccinate lyase	Inf	**Inf**
SAHH3	Adenosylhomocysteinase 3	2.76	**3.68**
TRXR3	Thioredoxin reductase 3	Inf	**Inf**
KAT3	Kynurenine–oxoglutarate transaminase 3	2.92	**4.24**
LDH	l-lactate dehydrogenase	1.54	**1.28**
GSTM3	Glutathione *S*-transferase Mu 3	2.9	**4.12**
CATA	Catalase	1.18	**2.12**
DHE3	Glutamate dehydrogenase	**1.23**	**1.68**

Significant differences (FDR < 0.05) are in bold.

## Discussion

The mud crab *S. paramamosain* is an osmoconformer in high salinity waters and an osmoregulator in low salinity waters^11^. In order to investigate osmoregulation and osmoconformation mechanisms of mud crabs, differentially expressed genes during acclimation to different salinities were profiled using transcriptomic and proteomic approaches here. We found a low correlation between expression levels of transcripts and proteins with only 31 differentially expressed genes in common at transcriptional and translational levels. The results are similar to findings from a previous study on salinity adaptation of mud crab^24^. Moreover, the poor correlation between transcript and protein abundance is also observed in other animal taxa^25,26^. The reason could be that post-transcriptional, translational and protein degradation regulation, other than transcript abundance, also affect protein abundance^27^. Yet the low correlation between transcriptomic and proteomic data allows us to understand salinity adaptation of mud crabs from different perspectives. The transcriptome data provide more evidence that ion transport and signal transduction genes participate in salinity adaptation of mud crabs, while proteomic data reveal more differentially expressed genes that are involved in energy metabolism and amino acid metabolism.

Our results show that four main categories of functional genes are involved in hyper-osmoregulation in the mud crab, including ion transport, signal transduction, energy metabolism and stress response. In contrast, amino acid metabolism and transport genes were upregulated at high salinity when the crabs exhibited osmoconformation, and few osmoregulatory genes showed differential expression in the process. Our results therefore indicate that osmoconforming crabs have a high capacity for osmolyte regulation, while osmoregulating crabs are capable of regulating ion transport actively. The roles of different categories of genes in osmoregulation and osmoconformation of the mud crab are elaborated below.

### Ion transport and signal transduction in osmoregulating crabs under low salinity

Ion transporters and channels are key components of osmoregulation. In the present study, upregulated DETs of low salinity groups are enriched in ion transport, supporting the critical roles of ion transport genes in hyper-osmoregulation. In contrast, few DETs of the high salinity groups are related to ion transport. Both transcriptomic and proteomic analyses show that ion transport related genes were upregulated in the low salinity groups, such as V-type H^+^ ATPase (VAT), carbonic anhydrase and chloride channel. Of them, VAT generates a H^+^ gradient across the apical membrane, which enables cations, such as Na^+^ to be transported into the cell via other transporters^28^, and thus critical for hyper-osmoregulation of crustaceans^29–31^. VAT usually shows elevated expression during low salinity stress in crustaceans, including the copepod *Eurytemora affinis*^31^, crabs *Portunus trituberculatus*^16^, *Eriocheir sinensis*^15^ and *Chasmagnathus granulatus*^32^, as well as the shrimp *Litopenaeus vannamei*^33^, indicating that its function is conserved among crustaceans. However, the responses of VAT to salinity stress are different among fishes, indicating its diverse function. For example, gill VAT activity increased in Atlantic salmon^34^ but decreased in rainbow trout^35^ exposed to low salinity waters. In contrast to crustaceans in which the estuarine and freshwater species originated from marine ancestors, fishes have historically moved between seawater and freshwater multiple times, resulting in highly diverse ion transport mechanisms (including VAT) among species^36,37^.

Some of the ion transport genes (e.g., AQ12A, G protein-activated inward rectifier potassium channel 4 and solute carrier family 26 member 6) that were differentially expressed in the transcriptomic analyses were not detected in the proteome, probably because their abundances are low in the mud crab gills. For example, transcripts of AQ12A showed downregulation under low salinity, similar with the results from studies on *P. trituberculatus*^17^ and barnacle *Balanus improvises*^38^. AQ12A is a member of the aquaporin family, which consists of water channels in cell membranes that mainly facilitate transport of water between cells^39^. Its downregulation indicates that mud crabs may decrease water uptake from the environment in order to avoid decrease of hemolymph osmolarity during low salinity acclimation. Interestingly, the expression of the Na^+^/K^+^ ATPase (NKA) remained unchanged in crab gills at all salinities tested in the present study, while it has been shown to play important roles in ion uptake of crustaceans and fishes, usually with increased expression under low salinity^11,16,29,33,40^. As NKA expression could vary with time^12,41^, a thorough study on its expression with more time points would help to elucidate its role in osmoregulation of the mud crabs. Nevertheless, we have identified a suite of ion transport genes involved in osmoregulation of crabs, similar to other decapods in general.

Besides ion transport, osmotic signal transduction is also important in the molecular mechanisms of osmoregulation in fish^19–21^, but this aspect is seldom explored in crustaceans. Although several studies touch upon osmotic signal transduction, they each focus on different genes^13,16,42,43^, such as integrin, crustacean hyperglycemic hormone and 14-3-3 like protein. Among these genes, only integrin showed differential expression in the present study. Integrins are transmembrane receptors for extracellular matrix components composed of an α and a β subunit, and key participants in osmotic signal transduction in fishes^21,44^. We found that ITAV (integrin alpha-V) was upregulated only in the 5 ppt/6 h group at both transcriptional and translational levels, and integrin-mediated signaling pathway (GO:0007229) was enriched in the 5 ppt/6 h group, supporting its participation in osmoregulatory signal transduction during early stage of low salinity adaptation in *S. paramamosain*.

In addition to integrin, we identified several other candidate genes involved in osmotic signal transduction, such as FAK1, HKR1 and SQH, which have not been reported to play this role in decapods. In detail, the gene FAK1 showed upregulation in the 5 ppt/6 h group (at transcriptional level). It was reported that the de-phosphorylation of focal adhesion kinase (FAK) can alter the activity of the Na^+^-K^+^-2Cl^−^ cotransporter and the cystic fibrosis transmembrane conductance regulator Cl^−^ channel in response to salinity change in fish^21^. Hence, as a member of the focal FAK family, FAK1 may contribute to hyper-osmoregulation in the mud crab in similar manner. Moreover, HKR1 (mucin-like transmembrane protein HKR1) transcripts showed increased expression only in the 5 ppt/6 h group, suggesting its potential role during osmotic signal transduction in mud crabs, considering it functions as osmosensor in yeast and animals^45,46^. However, the proteins of both FAK1 and HKR1 were not detected in this study, probably due to their low abundance. Besides, myosin regulatory light chain sqh (SQH) exhibited increased protein expression in the 5 ppt/6 h group. As SQH has been found to participate in cell volume regulation of mammals^47^, and also influences the Na^+^-K^+^-2Cl^−^ cotransporter activation in fish^48,49^, we speculate that it likely plays a role of mediating osmotic signal transduction in mud crab. Overall speaking, our results reveal several genes that may participate in signal transduction in salinity adaptation that warrant further investigation.

### Amino acid metabolism and transport in osmoconforming crabs under high salinity

Free amino acids serve as organic osmolytes that balance cellular osmotic pressure in osmoconforming crabs^4^. There are many researches showing that total free amino acid concentration in the gills of euryhaline crabs increases during high salinity acclimation^18,50,51^. Some amino acid metabolism genes and pathways show differential gene expression in response to salinity change^15,17,18^, but whether crabs use them to osmoconform or osmoregulate remains unknown. In the present study, mud crabs exhibited osmoconformation in response to high salinity stress. Also, genes related to amino acid metabolism are found to be upregulated during high salinity acclimation in mud crab. These genes are likely to function in osmoconformation by regulating production of free amino acids. For example, glutamate dehydrogenase is a potential control factor for the synthesis of proline and alanine, and has been found to participate in osmoconformation in decapods^18^ and copepods^52^. Aspartate aminotransferase participates in metabolism of alanine, arginine, cysteine and proline^53^. In short, a number of amino acid metabolism associated genes are found to be involved in osmoconformation of mud crabs.

Moreover, transcripts of two amino acid transport genes (i.e., EAA3 and MCATL) showed increased expression in the 33 ppt/6 h group. In particular, excitatory amino acid transporter 3 (EAA3) is a glutamate and aspartate transporter that plays an important role in glutamate and aspartate reabsorption in the human kidney^54,55^. This gene was upregulated in the 33 ppt/6 h group, suggesting it has a role in transporting amino acids from hemolymph into cells during the early stage of high salinity acclimation. In contrast, under low salinity exposure, although some amino acid metabolism genes also show differential expression, amino acid metabolism is not among the top enriched pathways in response to low salinity. This finding is consistent with results from previous studies^12,16^, suggesting amino acid metabolism plays a minor role in adaptation to low salinity as compared to its role in osmoconforming crabs in high salinity waters. In brief, through focusing on the molecular mechanisms of crab osmoconformation for the first time, we demonstrate the role of amino acid metabolism and transport genes in the process.

### Energy metabolism and stress response under low and high salinity

More energy is usually required when animals adapt to hypo-osmotic environment compared to iso-osmotic environment^56^, as active ion transport is energy consuming. To produce more energy animals metabolite more quickly, which could be characterized by increased food intake^57^, oxygen consumption^58–60^ and heart rate^61,62^. Both our transcriptomic and proteomic data show upregulation of energy metabolism genes in the gills of crabs exposed to low salinity waters, but not to high salinity waters. Under low salinity exposure, almost all differentially expressed genes associated with oxidative phosphorylation were upregulated, such as inorganic pyrophosphatase (IPYR) and NADH dehydrogenase. Our results demonstrate that the energy metabolism pathways of *S. paramamosain* are possibly boosted to cope with increased energy demand during hyper-osmoregulation. The finding is likely to hold true for most crustaceans, as energy metabolism associated genes also show upregulation under low salinity stress in the crabs *P. trituberculatus*^63^, *E. sinensis*^14,15^ and *Callinectes sapidus*^12^ as well as the shrimp *L. vannamei*^64^ and copepod *Lepeophtheirus salmonis*^65^. On the other hand, the expressions of energy metabolism genes showed no significant differences between crabs maintained at salinities of 25 ppt and 33 ppt. The result suggests that *S. paramamosain* does not require much extra energy during osmoconformation, at least within the range of salinities tested in this study. It has been reported in copepods that energy requirements remain unchanged during osmoconformation^66^, consistent to our findings. In all, it is evident that energy metabolism plays a major role in osmoregulating versus osmoconforming crabs.

Like other environmental stressors (e.g., high temperature and heavy metals), salinity is a stimulus for the production of heat shock proteins (HSP)^67,68^. Most of HSPs function as molecular chaperones to assist correct folding of new proteins or refold proteins damaged by cellular stress^67^. Many examples on the involvement of HSPs in hypo-osmotic stress have been documented in decapods, such as *L. vannamei*^69^, *Homarus americanus*^70^ and *E. sinensis*^15^, as well as copepods^65,71^ and fishes^68,72^, though the specific HSP genes often differ among studies. In our study, three HSP genes (ENPL, CH60 and HSP7C) were upregulated in the 5 ppt/6 h group, of which HSP7C was reported to participate in salinity stress response of *E. sinensis*^15^. The use of ENPL or CH60 as chaperones under salinity stress has not been reported in other decapods yet. No differential expression of HSP genes was detected between crabs exposed to salinities of 25 ppt and 33 ppt, indicating that high salinity is not as stressful as low salinity to the crab, or that the magnitude of salinity increase is too small to incite a stress response. To conclude, our results support the involvement of heat shock proteins in coping with low salinity stress in mud crab.

In summary, our results show, in a holistic way, the different pathways and mechanisms adopted by *S. paramamosain* during acclimation to low and high salinities. During osmoregulation under low salinity, osmotic signal transduction and stress response genes are mobilized within 6 h, and ion transport and energy metabolism genes are upregulated to actively uptake ions from the environment. Under high salinity, the crab achieves osmoconformation by producing more free amino acids, with the upregulation of amino acid metabolism genes. Mud crab cells may also increase the uptake of free amino acids from hemolymph at the early stage of osmoconformation. This study thus reveals the different mechanisms underlying osmoregulation and osmoconformation in mud crab, which broadly share among other euryhaline decapods and possibly crustaceans in general.

## Methods

Immature female *S. paramamosain* were purchased from Tai Po Market, Hong Kong. Three 150-L aquaria were used in the experiment with 15 individuals in each aquarium. Seawater was acquired from Tolo Habour, Hong Kong, and filtered before use. Freshwater was dechlorinated by aeration for a week. All crabs were acclimated at 25 ppt diluted seawater at 25 °C for a week. They were fed with fresh shrimp daily. Water was exchanged using a flow-through system at a flow rate of 3 L/min keeping NH_4_^+^  < 8 mg/L, NO_3_^−^ < 0.2 mg/L and a pH of about 8.2. Crabs were then acclimated to salinities of 5 ppt, 25 ppt or 33 ppt within 5 days. Three crabs in each treatment were sacrificed at 6 h, 1 day, 3 days and 10 days. Hemolymph was collected for osmolality measurements using a vapor pressure osmometer (Wescor, Logan, USA). Crabs at 25 ppt (control), at 5 ppt for 6 h (5 ppt/6 h) and 10 days (5 ppt/10 days), and at 33 ppt for 6 h (33 ppt/6 h) and 10 days (33 ppt/10 days) were used for transcriptomic and proteomic analyses, with three individuals in each treatment. As there were 15 crabs in total at 25 ppt, three individuals were selected randomly for analyses. The gills were isolated for total RNA and protein extraction. RNA-seq, real-time qPCR and LC–MS/MS proteomic analyses were subsequently conducted.

### Transcriptomic analyses

Total RNA was extracted using Invitrogen TRIzol reagent in accordance to the manufacturer’s protocol. The concentration of RNA samples was estimated using NanoDrop 2000 before samples were sent to Novogene (Beijing, China) for sequencing, where strand-specific TruSeq RNA library preparation and sequencing were conducted on an Illumina Hiseq X Ten platform. The Poly(A) option was chosen for mRNA enrichment. Raw sequencing reads were first evaluated by FASTQC v0.11.6^73^, and adaptors and low-quality reads were trimmed with TRIMMOMATIC v0.38^74^. Potential bacterial and viral contamination were filtered out with Kraken v1.0^75^. The resultant clean reads were de novo assembled using TRINITY v2.5.5^76^ and BinPacker v1.0^77^ separately. Transfuse v0.5 was then used to merge the two transcriptome assemblies (https://github.com/cboursnell/transfuse). BUSCO v3.0.2 was used to assess transcriptome assembly completeness by searching against the Arthropoda dataset^78^. For each transcript, the longest open reading frame was extracted using TransDecoder v3.0.0 implemented in Trinity^76^. All transcripts were searched against UniProt database using BLASTP with an E-value cutoff of 1e−5. Genes were tentatively identified according to the best hits against known sequences. KEGG database^79^ was used to infer the function of genes.

Differential gene expression analysis was performed using the Trinity pipeline for comparison among groups with three replicates. Transcript abundance was estimated with Kallisto v0.44.0, and Bowtie v2.3.4 was used to align transcripts^80,81^. Gene expression levels were normalized as transcripts per million transcripts (TPM). DESeq2 was used for differential expression analysis^82^, and the fold change cut-off was set at two-fold with FDR < 0.001. Differentially expressed genes (Figs. 2, 3 and Supplementary Fig. S2) were clustered using gplots package in R v3.4.4^83^. GO enrichment analysis of the differentially expressed genes was implemented using GOseq^84^ with a FDR < 0.05 cutoff. Real time qPCR was conducted on ten osmoregulation-associated genes for validation. The primers used (Supplementary Table S5 and S6) were designed online https://sg.idtdna.com/primerquest/Home/Index referring to transcripts generated by Transfuse above. A TaKaRa PrimeScript RT Reagent Kit (RR036B) was used for reverse transcription. TaKaRa TB Green Premix Ex Taq Kit (RR420A) was used for real time qPCR on a StepOne Real-Time PCR system. Each sample was analyzed three times. In order to choose an appropriate reference gene, the expression of six housekeeping genes were measured (Supplementary Table S6) and their stability was estimated with RefFinder^85^. HPRT (hypoxanthine–guanine phosphoribosyltransferase) had the highest stability and was used as the reference gene. Fold induction analysis was conducted using the ΔΔCt method.

### Label free proteomic analyses

Proteome analysis was conducted by integration of SDS-PAGE and LC–MS/MS^86,87^. Proteins in gill tissues were extracted using 8 M urea and a sonication method^86^, and then purified using a ReadyPrep 2D-cleanup kit (Bio-Rad, CA, USA). The concentration of proteins was quantified using a RC DC Protein Assay kit (Bio-Rad, CA, USA). For each sample, 100 μg of purified protein was used and separated in SDS-PAGE. The gel was stained using Blue-Silver Coomassie and then destained with 1% acetic acid. Each gel was divided into five fractions based on their intensity and molecular weight. Small gel pieces were further destained with 50% MeOH/50 mM NH_4_HCO_3_, MilliQ water, 100% acetonitrile (ACN), 100 mM NH_4_HCO_3_ and 100% ACN in order. Then, reduction and alkylation of proteins were conducted as described by Mu et al. and Ip et al.^86,87^. The protein samples were subsequently digested for 16 h at 37 °C using sequencing-grade 20 ng/µl trypsin in 50 mM NH_4_HCO_3_. Peptides were extracted from gel with 25 mM NH_4_HCO_3_, 5% formic acid in ACN, 100% ACN in order, and desalted using Sep-Pak C18 cartridges (Waters, Milford, USA). Five fractions of peptide samples were combined and dried in a vacuum concentrator (Eppendorf, Hamburg, Germany).

Dried peptides were reconstituted with 2% ACN in 0.1% formic acid, and then analyzed with Orbitrap Fusion Lumos Tribrid Mass Spectrometer (Thermo Fisher, Bremen, Germany). A PepMap C18 trap column (2UM 300UMx5MM NV 5PK) was used to separate peptides with 194 min LC gradient: 100% solution A (2% ACN in 0.1% formic acid) for 5 min, 0–6% solution B (98% ACN in 0.1% formic acid) for 8 min, 6–18% solution B for 48 min, 18–30% solution B for 58 min, 30–80% solution B for 60 min, maintained at 80% solution B for 5 min, and finally re-equilibrated at 100% solution A for 10 min. Mass spectrometry scans ranging from 375 to 1500 m*/z* were acquired with a resolution of 120,000 (at *m/z* 200). The data-dependent mode was selected for fragmentation in the high-energy collision-induced dissociation (HCD) cell with an isolation width of 0.7 *m/z*, and normalized collision energy of 38%. The Automatic Gain Control (AGC) target was set at 1e5 and maximum injection time at 105 ms. The dynamic exclusion time was 60 s, and the mass window for precursor ion selection was 10 ppm. Protein identification and quantitation were analyzed using MaxQuant v1.6.5.0 in Label-free Quantification (LFQ) mode with default settings^88^. A total of 68,564 putative protein sequences from transcriptome of *S. paramamosain* were set as reference database. One-way ANOVA was used for differential expression analysis and *p*-values were corrected for multiple testing by FDR method. Statistical significance was set at FDR < 0.05. Venn diagram was drawn online (https://bioinfogp.cnb.csic.es/tools/venny/index.html) to show the number of shared DETs or DEPs in the four treatment groups. To reveal the correlation of gene expression between transcriptional and translational levels, correlation analyses (Pearson and Spearman) and linear regression were performed for each experimental group based on transcripts per million reads (TPM) for transcripts and iBAQ (intensity based absolute quantification) for proteins.

## Supplementary information

Supplementary Information.

## Data Availability

Raw reads of RNA-seq were archived at NCBI SRA database with accession number PRJNA681094. Assembled transcriptomes were archived at NCBI TSA database with accession number GIXE01. All the MS data have been deposited to the ProteomeXchange Consortium via the PRIDE partner repository with the dataset identifier PXD022815.
