# Supplementary material for: Multi-omic approach provides insights into osmoregulation and osmoconformation of the crab Scylla paramamosain
Source: Sci Rep. 2020 Dec 10;10:21771. doi: 10.1038/s41598-020-78351-w (PMC7728780; doi:10.1038/s41598-020-78351-w)
Supplement: Supplementary file 1 — Supplementary Information. [file 41598_2020_78351_MOESM1_ESM.pdf]

Multi-omic approach provides insights into osmoregulation and osmoconformation of the crab *Scylla paramamosain*

Supplementary figures and tables

Jiaojiao Niu, Xue Lei Hu, Jack C.H. Ip, Ka Yan Ma, Yuan Yuan Tang, Yaqin Wang, Jing Qin, Jian-Wen Qiu, Ting Fung Chan and Ka Hou Chu

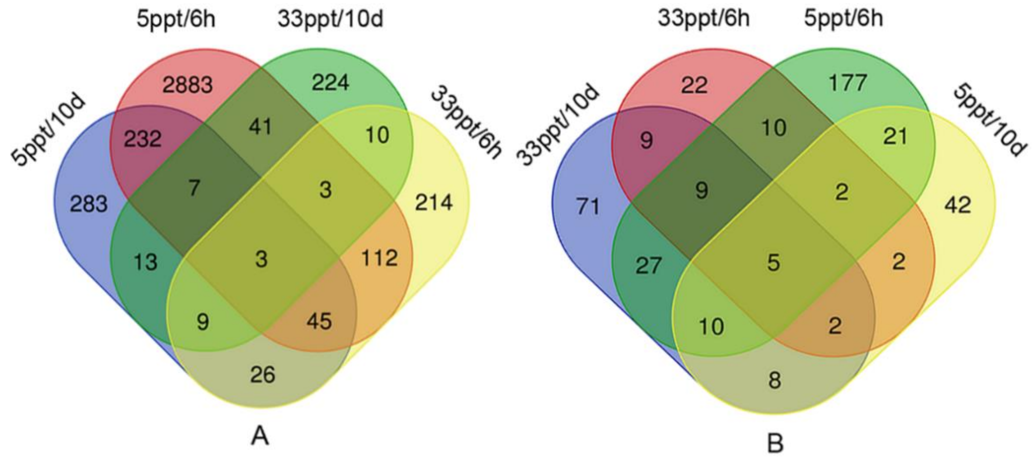

Figure S1. Venn diagram showing the number of differentially expressed transcripts (A) and proteins (B) in each treatment group.

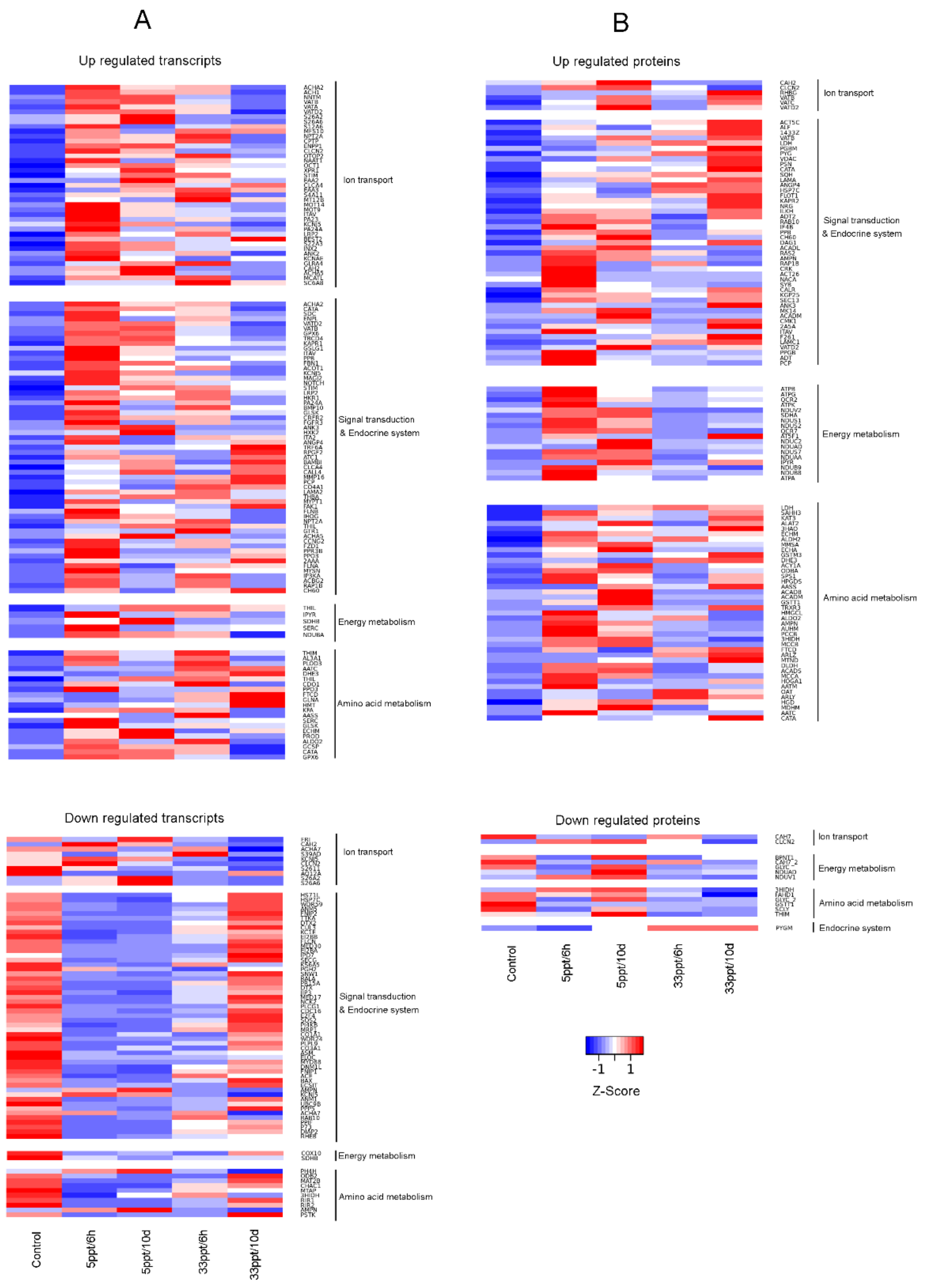

Figure S2. Heat map diagram of expression levels of differentially transcripts (A) and proteins (B) involved in ion transport, signal transduction, endocrine system, amino acid metabolism and energy metabolism using gplots package in R. High expression is indicated in red and low expression is indicated in blue. The columns represent control and treatment groups.

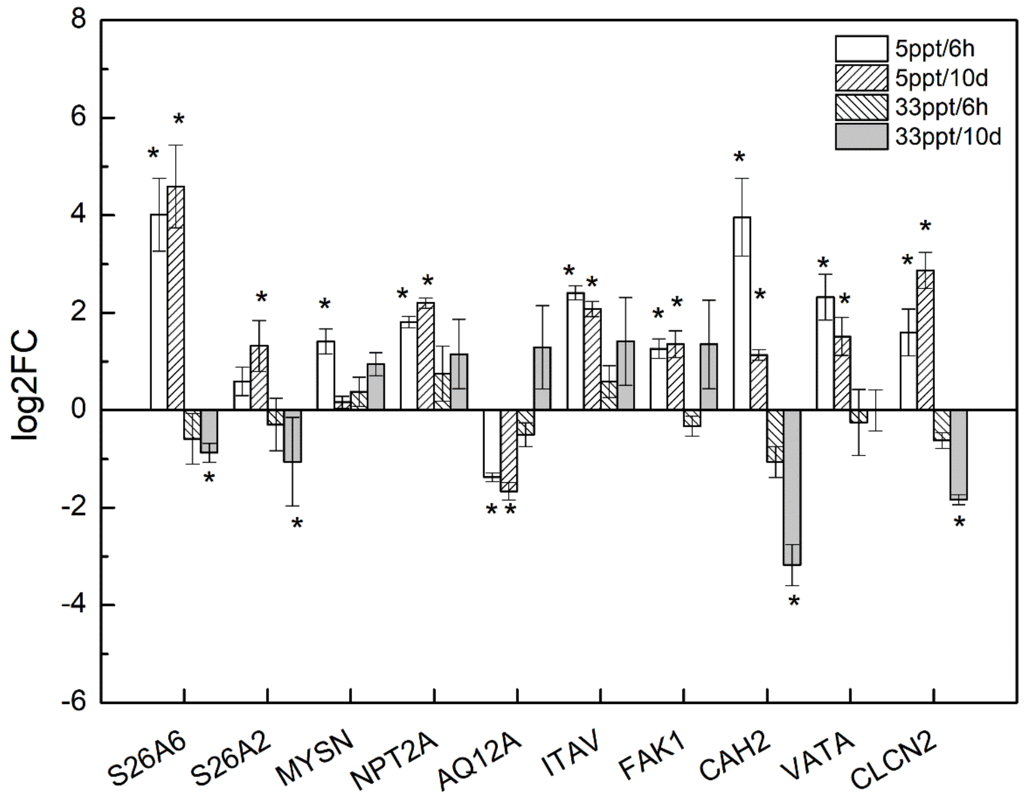

Figure S3. Log2 fold change of ten differently expressed genes detected by RT-qPCR. *Significant ($p < 0.05$, one-way ANOVA followed by Dunnett's multiple comparison) difference compared to control.

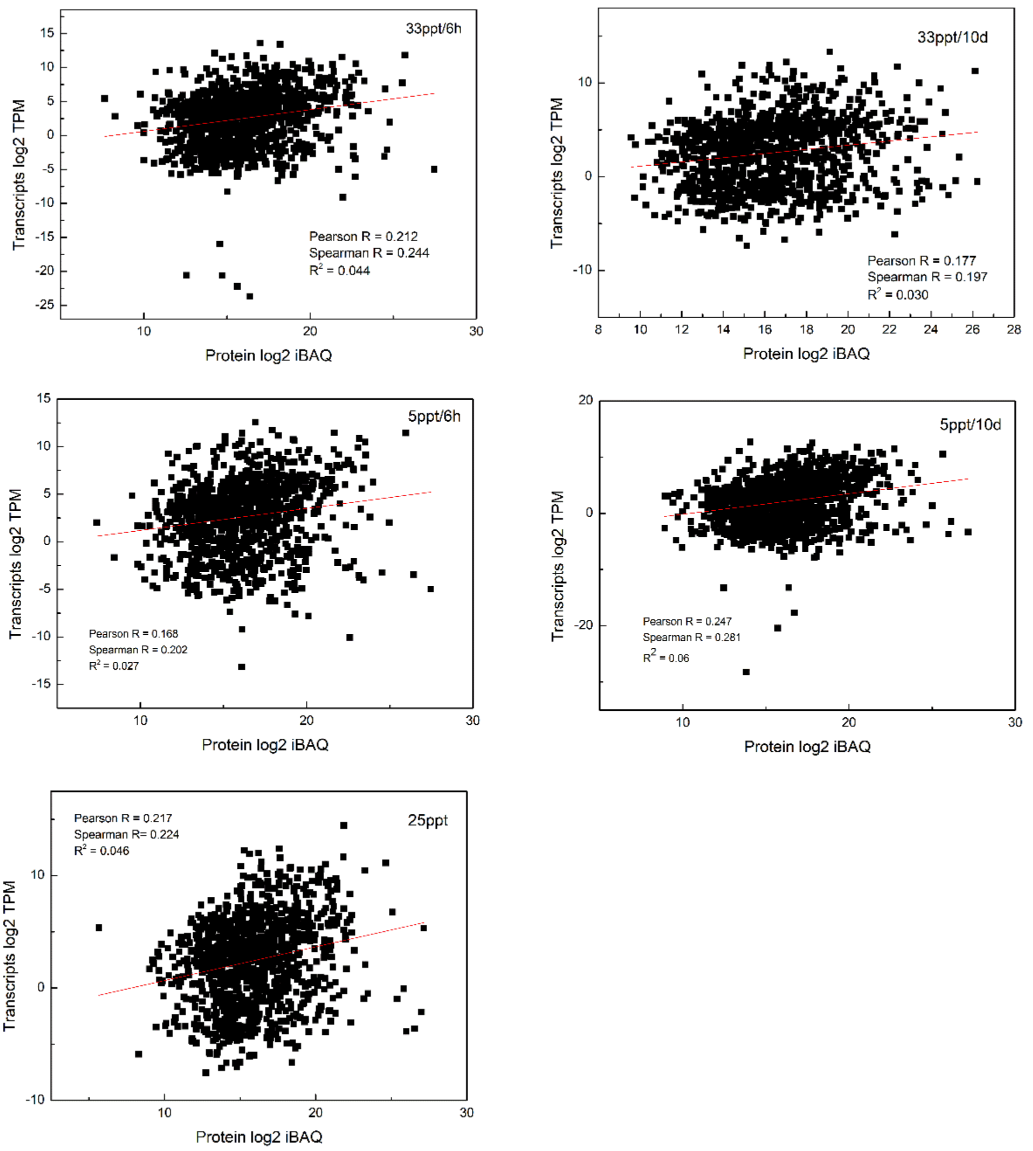

Figure S4. Correlation analyses on expression level of transcripts (log2 TPM) and proteins (log2 iBAQ) in each treatment group.

Table S1. Statistics and completeness of transcriptome assembly using different methods.

	Transcripts	N50	Average length	Total assembled bases	Complete BUSCOs	Completeness
BinPacker	133121	2123	1109.58	147708540	1041	97%
Trinity	374259	1365	754.94	282543728	1024	96%
Transfuse	257006	1396	836.56	214099927	1024	96%

Table S2. Number of differentially expressed (FDR < 0.001) transcripts and differentially expressed proteins (FDR < 0.05) in each treatment group.

Dataset	Treatment	Up-regulation	Down-regulation	Total
Transcripts	5ppt/6h	1543	1783	3326
	5ppt/10d	416	202	618
	33ppt/6h	237	185	422
	33ppt/10d	184	126	310
Proteins	5ppt/6h	204	56	260
	5ppt/10d	77	14	91
	33ppt/6h	41	19	60
	33ppt/10d	106	34	140

Table S3. Top ten significantly (FDR < 0.05) enriched GO terms of differentially expressed genes in each treatment group.

GO ID	p value	Number of DEGs	FDR	GO term
**5ppt/6h upregulation**				
GO:0005576	4.57E-14	50	9.26E-10	CC extracellular region
GO:0006811	8.00E-09	40	8.11E-05	BP ion transport
GO:0055062	2.99E-08	6	0.0001063	BP phosphate ion homeostasis
GO:0072506	2.99E-08	6	0.0001063	BP trivalent inorganic anion homeostasis
GO:0001871	3.23E-08	7	0.0001063	MF pattern binding
GO:0030247	3.23E-08	7	0.0001063	MF polysaccharide binding
GO:0072505	3.67E-08	6	0.0001063	BP divalent inorganic anion homeostasis
GO:0055083	4.19E-07	6	0.001062	BP monovalent inorganic anion homeostasis
GO:0006820	6.29E-07	22	0.0014163	BP anion transport
GO:0003974	1.16E-06	4	0.0023531	MF UDP-N-acetylglucosamine 4-epimerase activity
**5ppt/10d upregulation**				
GO:0006820	6.84E-09	12	0.0001385	BP anion transport
GO:0005310	2.34E-07	5	0.001736	MF dicarboxylic acid transmembrane transporter activity
GO:0015698	2.57E-07	6	0.001736	BP inorganic anion transport
GO:1902475	4.15E-07	4	0.0021028	BP L-alpha-amino acid transmembrane transport
GO:0008509	5.71E-07	10	0.0023147	MF anion transmembrane transporter activity
GO:0098656	8.06E-07	6	0.0023928	BP anion transmembrane transport
GO:0070777	1.04E-06	3	0.0023928	BP D-aspartate transport
GO:0070779	1.04E-06	3	0.0023928	BP D-aspartate import across plasma membrane
GO:0015711	1.47E-06	9	0.0023928	BP organic anion transport
GO:0015501	1.53E-06	3	0.0023928	MF glutamate:sodium symporter activity
**5ppt/6h downregulation**				
GO:0044428	5.81E-72	321	1.18E-67	CC nuclear part
GO:0032991	3.11E-61	370	3.15E-57	CC protein-containing complex
GO:0044424	6.28E-59	707	4.24E-55	CC intracellular part
GO:0044446	8.49E-53	471	4.30E-49	CC intracellular organelle part
GO:0044422	5.24E-51	474	2.12E-47	CC organelle part
GO:0044260	8.68E-51	407	2.93E-47	BP cellular macromolecule metabolic process
GO:0044464	1.61E-49	729	4.65E-46	CC cell part
GO:0043170	2.26E-49	424	5.74E-46	BP macromolecule metabolic process
GO:0006807	1.31E-42	443	2.66E-39	BP nitrogen compound metabolic process
GO:0043229	3.42E-41	488	6.30E-38	CC intracellular organelle
**5ppt/10d downregulation**				
GO:0006139	0	41	0	BP nucleobase-containing compound metabolic process
GO:0006725	0	41	0	BP cellular aromatic compound metabolic process
GO:0016070	0	34	0	BP RNA metabolic process
GO:0032991	0	45	0	CC protein-containing complex
GO:0034641	0	44	0	BP cellular nitrogen compound metabolic process

GO:0043170	0	48	0	BP macromolecule metabolic process
GO:0044260	0	46	0	BP cellular macromolecule metabolic process
GO:0044428	0	41	0	CC nuclear part
GO:0046483	0	41	0	BP heterocycle metabolic process
GO:0090304	0	39	0	BP nucleic acid metabolic process
**33ppt/6h downregulation**				
GO:0004377	1.49E-06	2	0.0092728	MF GDP-Man:Man3GlcNAc2-PP-Dol alpha-1,2-mannosyltransferase activity
GO:0019814	1.53E-06	3	0.0092728	CC immunoglobulin complex
GO:0042571	1.53E-06	3	0.0092728	CC immunoglobulin complex, circulating
GO:0034987	1.83E-06	3	0.0092728	MF immunoglobulin receptor binding
GO:0050853	3.35E-06	3	0.0135837	BP B cell receptor signaling pathway
GO:0033577	4.95E-06	2	0.0167216	BP protein glycosylation in endoplasmic reticulum
GO:0006958	7.03E-06	3	0.0177829	BP complement activation, classical pathway
GO:0004376	7.42E-06	2	0.0177829	MF glycolipid mannosyltransferase activity
GO:0006910	7.90E-06	3	0.0177829	BP phagocytosis, recognition
GO:0000026	1.04E-05	2	0.0210499	MF alpha-1,2-mannosyltransferase activity
**33ppt/10d downregulation**				
GO:0031508	1.31E-10	4	2.66E-06	BP pericentric heterochromatin assembly
GO:0031055	7.67E-10	4	7.77E-06	BP chromatin remodeling at centromere
GO:0031507	1.95E-09	4	1.32E-05	BP heterochromatin assembly
GO:0070828	2.95E-08	4	0.0001492	BP heterochromatin organization
GO:0031497	6.65E-08	4	0.0002697	BP chromatin assembly
GO:0006333	4.63E-07	4	0.0015623	BP chromatin assembly or disassembly
GO:0006821	1.08E-06	4	0.002357	BP chloride transport
GO:0008271	1.27E-06	3	0.002357	MF secondary active sulfate transmembrane transporter activity
GO:0019531	1.44E-06	3	0.002357	MF oxalate transmembrane transporter activity
GO:0015116	1.67E-06	3	0.002357	MF sulfate transmembrane transporter activity

Table S4. Genes showed differential expression in both transcriptomic and proteomic data. Upregulated genes were indicated with + while downregulated genes were indicated with -.

Gene ID	Gene name	5ppt/6h	5ppt/10d	33ppt/6h	33ppt/10d	KEGG function
F16P1	Fructose-1,6-bisphosphatase 1	+				Carbohydrate metabolism
NNTM	NAD(P) transhydrogenase	+				Oxidoreductases
VATB	V-type proton ATPase subunit B	+				Energy metabolism, ion transport
ST1B1	Sulfotransferase family cytosolic 1B member 1	+				Transferases
PCP	Lysosomal Pro-X carboxypeptidase	+				Protein digestion and absorption
60A	Protein 60A	+				N/A
RDL	Putative thiosulfate sulfurtransferase	+				Energy metabolism
ALDO2	Indole-3-acetaldehyde oxidase	+				Amino acid metabolism
PCE	Proclotting enzyme	+				N/A
LACH	Lachesin	+				N/A
FLNA	Filamin-A	+				Cytoskeleton proteins, signal transduction
VATD2	Probable V-type proton ATPase subunit D 2	+				Energy metabolism, ion transport
RAP1B	Ras-related protein Rap-1b	+				Signal transduction
ITAV	Integrin alpha-V	+				Signal transduction
IPYR	Inorganic pyrophosphatase	+	+			Energy metabolism
CH60	60 kDa heat shock protein, mitochondrial	+				Stress response
PPB	Alkaline phosphatase	+				Signal transduction
PTGR1	Prostaglandin reductase 1	-				Oxidoreductases
RLA2	60S acidic ribosomal protein P2	-				Genetic information Processing
RDRP	RNA-directed RNA polymerase VP1	-		-		Genetic information Processing
RL8	60S ribosomal protein L8	-				Genetic information Processing
FRRS1	Putative ferric-chelate reductase 1 homolog		+			N/A
ECHM	Enoyl-CoA hydratase		+	+		Amino acid metabolism
CLCN2	Chloride channel protein 2		+		-	Ion transport
CAH2	Carbonic anhydrase 2		+			Energy metabolism
NXN	Nucleoredoxin			+		Protein phosphatases and associated proteins
EST6	Venom carboxylesterase-6			+		Lipid metabolism
FTCD	Formimidoyltransferase-cyclodeaminase			+	+	Amino acid metabolism

DHE3	Glutamate dehydrogenase	+	+	Amino acid metabolism
TBA	Tubulin alpha chain		+	Cytoskeleton proteins
CUPC3	Cuticle protein CP1246		+	N/A

Table S5. Primers of 10 target genes of *S. paramamosain* for quantitative real-time PCR.

Gene name	Gene ID	Primer L	Primer R	Tm
V-type proton ATPase subunit A	VATA	CTCAGGCACTGTCCAAGTATTC	CCTTCTTACCGTTCACCTCAAG	62
Carbonic anhydrase 2	CAH2	TTCCCTTCATCAGCCACAAG	GAGCCGCTGTAAGTGTAGTATG	62
Chloride channel protein 2	CLCN2	CGCAGGAACAAGAGGATGAA	GGACACGGACCCATAAAGTAAG	62
Aquaporin-12A	AQ12A	GGGTGAACTCCGACCTAAGTAT	GGTTAAAGTAGCCGCCAGAATAG	61
Sodium-dependent phosphate transport protein 2A	NPT2A	CAATGCTGAGGTTCCCTATGT	GGAGTGAGTGTTGAGGTGAAA	61
Integrin alpha-V	ITAV	CGGGTTCTCATCAACACGTAA	TCAGGGACTCAAATGGACAAC	61
Myosin heavy chain, non-muscle	MYSN	GAACCCGCCCAAATTCAATAAG	TGTAGATCAGGCCGGAGTAATA	61
Focal adhesion kinase 1	FAK1	CATCCAAGGGAAAGCTACCTATTA	CATGCACACACCAAACATCC	61
Sulfate transporter	S26A2	ACCTGGCTGCCTACCTATAA	GGAATATGCATGATGGCAACAG	61
Solute carrier family 26 member 6	S26A6	GTGGATGCTACTCAGGAACTTTA	GATCAATGAGCGAGACAGAGAG	65

Table S6. Primer sequence and stability of six housekeeping genes

Gene name	Gene ID	Primer L	Primer R	Tm	Stability
Hypoxanthine-guanine phosphoribosyltransferase	HPRT	CTGGCCATCCTGCAGAAATA	CACAGTAATCCGGCCTGTATC	62	1.19
Glyceraldehyde-3-phosphate dehydrogenase	G3P	ACCACTGTTCATGCTGTTACT	GGAGGATGGGATGATGTTCTG	62	2.51
TATA-box-binding protein	TBP	CCACAGTCAGTTCAGCAGTTA	CACCTTGCCAGACACGAATA	62	5.73
Succinate dehydrogenase flavoprotein subunit	SDHA	TGCAGTTTCATCCTACAGGTATC	CAGGCGCATATCTCTCCATAAA	62	4.95
Tubulin beta-2 chain	TBB2	TGGTTGAGAACACCGATGAG	CCATAGGTTGGGTTCTGTAGTT	62	3.46
Beta actin	ACT	GAGAAGATCTGGCACCACACTTT	CGCGGTTGGCCTTGG	62	2.06
